# Stressors and Pain across the Late-Life Span: Findings from Two Parent Longitudinal Studies of Aging and Health

**DOI:** 10.1177/08982643221104369

**Published:** 2022-06-06

**Authors:** Penny L. Brennan

**Affiliations:** 1 Institute for Health & Aging, 8785 University of California San Francisco, San Francisco, CA, USA

**Keywords:** stressors, pain, older adults, longitudinal

## Abstract

**Objective:**

The objective is to determine associations between stressors and pain across the late-life span.

**Method:**

Multilevel linear modeling was applied separately to harmonized repeated measures data from the Longitudinal Late-Life Health study (LLLH; *n* = 342; 13-year interval) and the Health and Retirement Study (HRS; *n* = 2959; 8-year interval).

**Results:**

In both the LLLH and HRS samples, independent of age, gender, and race, participants with higher average stressor levels experienced more numerous painful conditions and higher pain severity over the study intervals. In the HRS sample, they also experienced higher levels of pain interference. In general, participants’ stressor levels did not influence rates of increase in their pain. Gender and race had few moderating effects on associations between stressors and pain.

**Discussion:**

Stressors and pain are associated across the late-life span. Future research should focus on the mediating mechanisms that account for this association and the moderating factors that affect its strength.

## Introduction

Pain is common in later life. Between 53% and 66% of late-middle-aged and older adults report experiencing pain in the last month (Patel et al., 2013; Scudds & Ostby, 2001; Thomas et al., 2004); 72% to 86% of older adults report having had pain in the last year (Brattberg et al., 1996; Brochet et al., 1998; Mobily et al., 1994). Almost 40% of older adults report chronic pain, pain persisting for the last 3–12 months (Cimas et al., 2018; Larsson et al., 2017). Chronic and other forms of pain may increase, or reach peak levels, during later life (Abdulla et al., 2013; Ahacic & Kåreholt, 2010; Fayaz et al., 2016), and pain prevalence among older adults appears to be growing (Zimmer & Zajacova, 2020). This high and increasing pain prevalence among older adults underscores the importance of further research aimed at the prediction, prevention, and effective treatment of late-life pain (American Geriatrics Society, 2009; Institute of Medicine, 2011; Briggs et al., 2016; Cimas et al., 2018; Larsson et al., 2017; Patel et al., 2010; Zimmer & Zajacova, 2020).

Most pain and aging research is based on the biopsychosocial model of pain, which posits that pain is a product of complex interactions among biological, psychological, and social factors (e.g., Edwards et al., 2016; Gatchel et al., 2007; Turk et al., 2016). However, relatively little research has focused on the *social* component of this model. Some recent studies of mixed-age samples have demonstrated the influence of social environmental factors, including early-life trauma (Afari et al., 2014; Edwards et al., 2016; Kopec & Sayre, 2004, 2005), financial hardship (Borsook et al., 2018), neighborhood disorder (Ulirsch et al., 2014), adverse workplace conditions (Edwards et al., 2016; Helmhout et al., 2010; Melloh et al., 2013; Pincus et al., 2013), and low social support (Edwards et al., 2016; Jensen et al., 2011), on pain. In several of these studies, the explanatory factor for poorer pain outcomes is conceived to be *“stress”* (Buscemi et al., 2019), the intrapersonal, psychological, and/or physiological experience indicative of being taxed by environmental conditions (Crosswell et al., 2020; Lazarus & Folkman, 1984). However, stress is distinguishable from *stressors*, the environmental events or circumstances that tax one’s functioning and well-being (Crosswell et al., 2020; Wheaton et al., 2013). Sociology of health research has substantiated that stressor elevations are associated with negative health outcomes across a range of major medical conditions, including cardiovascular illness, rheumatoid arthritis, and cancer, and with more depressive symptoms, among people of all ages (Crosswell et al., 2020; McEwen, 1998; Slavich, 2016; Thoits, 2010). Thus, stressors might also be expected to affect older adults’ experience of pain.

Consistent with this expectation, a recent cross-sectional study (Brennan, 2020) showed that independent of their age, gender, and race, older adults reporting elevated stressors in the domains of negative life events, early-life trauma, neighborhood, work, finances, spouse, children, extended family, and friends, and in stressors overall, were at heightened risk of reporting joint, back, headache, and chest pain, and having more numerous painful conditions, more severe pain, and more pain interference. Stressor exposure was higher for men than women in most stressor domains, and it was higher for non-Whites than Whites in all stressor domains. Pain was more prevalent among women and non-Whites than men and Whites. For certain domains of stressors, and types of pain, there were some interactions between pain and gender, and pain and race, to predict stressor elevations, which provided some, but very limited, support for the *differential vulnerability hypothesis* (Aneshensel, 1992; Kessler, 1979), the proposition that the strength of the association between stressors and poor health outcomes are stronger among women than men and among non-Whites compared to Whites. In Brennan (2020), findings generally reproduced across separate samples of older adults drawn from two large parent longitudinal studies of aging and health.

The present study expands upon that earlier work to adopt a longitudinal, late-life course perspective on older adults’ stressors and pain. This approach aligns broadly with the use of a life-course framework (Pearlin, 2010) to study aging and health. The life-course framework assumes that older adults are enveloped by, and must adapt to, changing environmental circumstances, such as stressor fluctuations, over the course of later life. It posits that stressors experienced over the course of later life, and older adults’ responses to them, are potent determinants of older adults’ late-life health and symptom trajectories.

A longitudinal, life-course perspective also aligns with the practice in contemporary pain research of conceptualizing and measuring individuals’ pain experiences as *trajectories*, obtained through multiple longitudinal assessments, across long time intervals. These pain severity and pain interference trajectories are favored in pain research because they better capture peoples’ pain experience than do single time-point pain “snap shots.” Further, these trajectories have the benefit of providing clinicians with a longer prognostic perspective on their patients’ pain (Dunn, 2010; Kongsted et al., 2016).

Adoption of a longitudinal, life-course perspective on late-life stressors and pain is made possible by the availability of data from separate large, longitudinal studies of aging and health in which older adults’ stressors and pain have been assessed using the same or very similar measures, repeatedly, over relatively long time intervals. Stressors and pain measures can be harmonized across these separate data sources, and then the data examined to determine whether hypothesized relationships between stressors and pain found in one of the longitudinal studies of aging and health can be reproduced in the other. Findings reproduced across two samples in this way can enhance confidence in the veracity of hypothesized relationships among constructs in theoretical models of late-life stressors and pain.

The purpose of this study is to adopt a longitudinal, life-course perspective to determine associations between stressors and pain across the late-life span. Earlier, cross-sectional findings (Brennan, 2020) provide bases for the hypotheses that: (1) Older adults who experience higher levels of stressors over the late-life course will also experience higher levels of, and faster rates of increase in, number of painful conditions, pain severity, and pain interference during the late-life span; (2) Gender and race will have little moderating influence on this relationship; and (3) These findings will replicate across samples drawn from two separate, parent longitudinal studies of aging and health, whose participants have been assessed with harmonized, repeated measures of stressors and pain.

## Method

### Samples

The samples for this investigation were drawn from two parent investigations of aging and health: Longitudinal Late-Life Health (LLLH) and the Health and Retirement Study (HRS).

Longitudinal Late-Life Health (LLLH) was a 20-year longitudinal study of 1,884 late-middle-aged community residents from Northern California. Data for this investigation were collected through mailed surveys and interviews, for a total of six waves of data collection, the first beginning in 1986–1988 and the last occurring in 2007–2009. At baseline assessment, LLLH participants were between the ages of 55 and 65, almost 90% White, and comparable to similarly aged national community samples with respect to health characteristics. (For further details on the LLLH baseline sample, see Brennan & Moos, 1990; Moos et al., 1990.) Data regarding pain were collected from a partial sample of participants (*n* = 689), beginning at the 1996 data collection. The LLLH longitudinal sample examined here comprises the *n* = 342 individuals who provided data regarding their stressors and pain at each of the last three measurement points in the LLLH investigation: 1996, 1999, and 2009.

The Health and Retirement Study (HRS) has conducted longitudinal biennial health assessments (“core interviews”) of nationally representative samples of adults age 50+ since 1992 (Health and Retirement Study, 2022; Servais, 2010; Sonnega, 2015; Smith et al., 2017). In 2006, HRS began for the first time to collect psychosocial data from study participants, including information about their stressors in the domains of childhood/adolescence trauma, neighborhood environment, work, finances, and interpersonal relationships with spouses, children, extended family members, and friends (Smith et al., 2017). The HRS psychosocial questionnaire is administered to HRS participants every 4 years post-baseline assessment. Thus, the HRS longitudinal sample for this investigation comprises the 2,959 older adults who completed the HRS psychosocial questionnaire and core interview, and thus provided data regarding their stressors and pain, at each of the three measurement points: 2006, 2010, and 2014.

### Measures

Baseline demographic characteristics. In both the LLLH and HRS samples, baseline measures of demographic characteristics included *age*, in years, and *gender* (0 = male; 1 = female). The LLLH and HRS samples were predominately White (91% and 83%, respectively). Due to this very skewed distribution, *race* was dichotomized as 0 = White and 1 = non-White.

Stressors. Five stressor measures were chosen for use in this study: financial stressors, and interpersonal stressors in the domains of spouse, children, extended family, and friends. These stressor measures were chosen because they were used in both the LLLH and HRS parent investigations at all three measurement points: 1996, 1999, and 2009 (LLLH) and 2006, 2010, and 2014 (HRS). With the exception of financial stressors in the HRS investigation, all of these stressor measures were subscales of the Life Stressors and Social Resources Inventory (LISRES; Moos & Moos, 1994; Moos, 2011). They have been shown to have good reliability and validity (Brennan & Moos, 1990; Moos, 2011; Moos & Moos, 1994; Smith et al., 2017).

In the LLLH sample, *financial stressors* were assessed with a sum of responses to the 6 items from the LISRES (“Do you have enough money to afford...”, for example, “a large unexpected bill”; “adequate food and clothing”) rated on a scale of 0 (definitely no) to 3 (definitely yes). In the HRS sample, they were assessed with the Campbell et al. (1976) measure of financial strain, the item “How difficult is it for you to meet your monthly payments?”, rated on a scale of 0 (not at all) to 4 (very difficult).

In both the LLLH and HRS samples, *interpersonal stressors* were measured with the same 6 items from the LISRES, repeated in each four life domains (spouse, children, extended family, and friends), that assess how much the target person (e.g., spouse) is critical, cannot be relied upon, makes too many demands, gets on your nerves, does not understand how you feel, and lets you down. In the LLLH sample, participants rated interpersonal stressor items on a scale of 0–4, ranging from 0 = never to 4 = often. In the HRS sample, these items were rated on a scale of 0–3, ranging from 0 = not at all to 3 = a lot. For both samples, a composite measure of *overall stressors* was calculated by summing participants’ stressor scores across the five financial and interpersonal stressor domains, then dividing the sum by the number of non-missing domains. This was done because certain stressor domains were not applicable for some participants. For example, some participants were not partnered due to divorce or widowhood; accordingly, they did not provide information about spouse stressors. Participants’ scores on this measure ranged from 0 to 13, with higher scores indicating more overall stressors.

Pain. In both the LLLH and HRS samples, *number of painful conditions* was a summation of the presence (0 = no and 1 = yes) of each of four types of pain: joint, back, headache, and chest. In the LLLH study, all participants provided ratings of *pain severity* and *pain interference*, which were assessed with the two SF-36 Health Survey (Ware et al., 1998) items: “How much bodily pain have you had in the past month?” (rated 0 = none to 5 = very severe), and “During the past month, how much did pain interfere with your normal activities (including activities inside and outside your home)?” (rated 0 = not at all to 4 = extremely).

In the HRS study, participants were asked whether they were often troubled by pain; those answering affirmatively were asked to rate their *pain severity* on a scale of 0 = none to 3 = severe, and *pain interference* (whether pain made it difficult to engage in usual activities; 0 = no and 1 = yes). For participants who did not answer affirmatively, zero values were imputed for pain severity and pain interference.

### Statistical Analyses

Statistical analyses were conducted separately on LLLH and HRS sample data. First, descriptive statistics were conducted to determine participants’ baseline age, gender, and race. Then, multilevel linear modeling was conducted to estimate unconditional linear growth models of levels, and rates of change, in participants’: (a) overall stressors, and stressors within the domains of finances, spouse, children, extended family, and friends; and (b) pain, including number of painful conditions, pain severity, and pain interference. Next, multilevel linear modeling was used to estimate conditional growth models in which the predictor was participants’ cross-time, overall stressors levels, and the outcomes were participants’ overall levels, and rates of change in, number of painful conditions, pain severity, and pain interference, over the 13-year (LLLH) and 8-year (HRS) time intervals spanned in this investigation. Baseline age, gender, and race were included as covariates in each of these conditional growth models. Finally, multilevel linear modeling was used to first determine interactions between gender and six categories of stressors (finances, spouse, children, extended family, friends, and overall stressors), on levels and rates of change in, three pain outcomes (number of painful conditions, pain severity, and pain interference). These analyses were then repeated to determine interactions between race and stressors on levels and rates of change in the three pain outcomes. SPSS 26 statistical software was used to conduct the analyses in this investigation.

## Results

### Unconditional Growth Models of Stressors

Table 1 shows unconditional growth models of the levels and rates of change in stressors in the LLLH 13-year and HRS 8-year longitudinal samples. In the unconditional growth model for LLLH participants, the estimated average level of stressors overall was 6.17 (*p* < .01), with a within-person average rate of decline in overall stressors of −.08 (*p* < .01). Among HRS participants, the estimated average level of stressors overall was 3.71 (*p* < .01), with a within-person average rate of decline in stressors of −.18 (*p* < .01).

**Table 1.** Unconditional Growth Models of Stressors in the LLLH and HRS Samples.

	Overall stressors		Financial stressors		Spouse stressors		Child stressors		Extended family stressors		Friend stressors	
	LLLH	HRS	LLLH	HRS	LLLH	HRS	LLLH	HRS	LLLH	HRS	LLLH	HRS
Fixed effects												
Initial status	6.17^**^	3.71^**^	2.76^**^	1.94^**^	5.84^**^	4.69^**^	6.49^**^	4.14^**^	9.03^**^	4.47^**^	6.95^**^	3.55^**^
Rate of change	−.08^**^	−.18^**^	.08	−.07^**^	.06	−.06	−.32^**^	−.23^**^	−.24^*^	−.21^**^	.06	−.12^**^
Variance components												
In initial status	4.01*^*^	2.64^**^	12.92^**^	.74^**^	12.81^**^	8.27^**^	13.81**	8.14**	8.53^**^	6.68^**^	5.70^**^	4.98^**^
In rate of change	.20	.16^**^	1.12**	.05^**^	.30	.47**	1.29**	.50**	.32	.38**	.59	.32**
Covariance	−.37	−.31**	−2.44**	−.11**	−1.06	−.76**	−2.87**	−.98**	−.60	−.84**	−.86	−.83**
Residual	1.97**	1.01**	3.99^**^	.30**	5.67^**^	3.47^**^	5.13^**^	3.28^**^	7.14^**^	4.12^**^	4.95**	2.72*^*^
Model fit												
−2 LL deviance	4289.5	31,873.2	5131.6	20,603.6	3938.3	28,315.1	4850.6	38,665.6	4850.6	39,559.2	5064.7	36,032.9
AIC	4301.5	31,885.2	5143.7	20,615.6	3950.3	28,321.1	4862.6	38,667.6	4862.6	39,579.8	5076.7	36,044.9
BIC	4331.1	31,927.8	5173.2	20,658.1	3978.0	28,367.1	4891.6	38,719.6	4891.6	39,613.9	5106.2	36,087.1

*Note.* LLLH = Longitudinal Late-Life Health sample; HRS = Health and Retirement Study sample; −2LL = −2 log likelihood; AIC = Akaike’s Information Criterion; BIC = Schwarz’s Bayesian Criterion.

* *p* < 0.05; ** *p* < 0.01.

With respect to the five individual stressor domains that comprised the overall stressor measure, participants in both the LLLH and HRS samples experienced declines in child (−.32, *p* < .01, and −.23, *p* < .01, respectively) and extended family stressors (−.24, *p* < .05, and −.21, *p* < .01, respectively). Whereas HRS participants experienced declines in stressors involving finances (−.07, *p* < .01) and friends (−.12, *p* < .01), this was not the case among the LLLH participants. Neither LLLH nor HRS participants experienced declines in spouse stressors.

### Unconditional Growth Models of Pain

Unconditional growth models of pain in the longitudinal LLLH and HRS samples are shown in Table 2. In these models, baseline average levels of number of painful conditions, pain severity, and pain interference were relatively low but increased significantly over time in both the LLLH and HRS samples. On average, at baseline, LLLH and HRS participants had one painful condition (.80, *p* < .01; 1.16, *p* < .01) and experienced a slight increase in number of painful conditions (.15, *p* < .01; .03, *p* < .01) over the ensuing 13 and 8 years. In the LLLH and HRS samples, average overall levels of pain severity were relatively low (1.52, *p* < .01; .50, *p* < .01) but increased over time (.21, *p* < .01; .07, *p* < .01). Similarly, pain interfered minimally with LLLH and HRS participants’ activities at initial assessment but did so increasingly over the next 13 years (.20, *p* < .01; LLLH) and 8 years (.03, *p* < .01; HRS).

**Table 2.** Unconditional Growth Models of Pain in the LLLH and HRS Samples.

	Number of painful conditions		Pain severity		Pain interference	
	LLLH	HRS	LLLH	HRS	LLLH	HRS
Fixed effects						
Initial status	.80^**^	1.15^**^	1.52^**^	.50^**^	.50^**^	.15^**^
Rate of change	.15^**^	.03^**^	.21^**^	.07^**^	.20^**^	.03^**^
Variance components						
In initial status	.69^**^	.80^**^	1.32**	.45^**^	1.95^**^	.06^**^
In rate of change	.04	.04**	.08	.03**	.28**	.01**
Covariance	−.11	−.12**	−.23*	−.04*	−.60**	.00
Residual	.49^**^	.21^**^	.88^**^	.44^**^	.32^**^	.09^**^
Model fit						
−2 LL deviance	2657.4	18,291.9	3261.3	22,399.0	2757.8	7590.8
AIC	2669.4	18,303.9	3273.4	22,441.0	2769.8	7602.8
BIC	2698.9	18,346.4	3302.9	22,453.6	2799.4	7645.3

*Note.* LLLH = Longitudinal Late-Life Health sample; HRS = Health and Retirement Study sample; −2LL = −2 log likelihood; AIC = Akaike’s Information Criterion; BIC = Schwarz’s Bayesian Criterion.

* *p* < 0.05; ** *p* < 0.01.

Statistically significant variability in the initial status variance component of these unconditional growth models of pain show potential for stressors to predict average levels of pain over 13 years and 8 years in the LLLH and HRS samples. The rate of change variance component shows the potential for stressors to predict growth over time in pain interference in the LLLH sample, and growth over time in all three of the pain indices in the HRS sample.

### Effects of Stressors on Levels and Rates of Change in Pain

Table 3 summarizes the results of conditional growth models wherein stressors predict levels and rates of change in number of painful conditions, pain severity, and pain interference in the LLLH and HRS samples. Independent of age, gender, and race, higher levels of stressors overall, spanning 13- year (LLLH) and 8-year (HRS) intervals, were associated with experiencing more numerous painful conditions over those intervals (.10, *p* < .01 in the LLLH sample; .04, *p* < .01 in the HRS sample). In both the LLLH and HRS samples, elevations of spouse stressors, and of child stressors, were associated with more painful conditions (.06, *p* < .01 and .02, *p* < .01, respectively). Only in the LLLH sample, heightened friend stressors were associated with more numerous painful conditions (.05, *p* < .05). Only in the HRS, more financial stressors were linked to having more numerous painful conditions (.09, *p* < .01).

**Table 3.** Effects of Stressors on Levels and Rates of Change in Number of Painful Conditions, Pain Severity, and Pain Interference in the LLLH and HRS Samples.

NUMBER OF PAINFUL CONDITIONS									
	Fixed effects		Variance components				Model fit		
	Intercept	Rate of change	In intercept	In rate of change	Covariance	Residual	−LL	AIC	BIC
Overall stressors									
LLLH	.10^**^	−.02	.56^**^	.03^**^	−.07^**^	.49^**^	2623.4	2651.4	2720.5
HRS	.04^**^	−.01^*^	.77^**^	.04^**^	−.12^**^	.21^**^	18,183.0	18,211.0	18,310.1
Financial stressors									
LLLH	.00	.01	.66^**^	.03	−.10	.49^**^	2634.7	2662.7	2731.8
HRS	.09^**^	−.01	.77^**^	.04^*^	−.12^**^	.21^**^	17,906.9	17,934.9	18,033.8
Spouse stressors									
LLLH	.06^**^	−.02	.62^**^	.02	−.07	.45^**^	1917.2	1945.2	2009.9
HRS	.02^**^	−.00	.76^**^	.05^**^	−.12^**^	.21^**^	12,248.6	12,276.6	12,369.9
Child stressors									
LLLH	.06^**^	−.01	.44^**^	.01	−.03	.51^**^	2401.8	2429.8	2497.6
HRS	.02^**^	−.00	.79^**^	.04^**^	−.12^**^	.21^**^	16,754.9	16,782.9	16,880.8
Family stressors									
LLLH	.03	−.01	.48^**^	.00	−.02	.51^**^	2434.7	2462.7	2530.7
HRS	.01^*^	−.01^*^	.79^**^	.04^**^	−.12^**^	.21^**^	16,971.2	16,999.2	17,097.2
Friend stressors									
LLLH	.05^*^	−.01	.65^**^	.04	−.11	.49^**^	2626.0	2654.0	2723.0
HRS	.01	−.00	.81^**^	.05^**^	−.13^**^	.20^**^	18,448.9	18,454.9	18,476.2
PAIN SEVERITY									
Overall stressors									
LLLH	.11^**^	−.01	1.12^**^	.07	−.19^**^	.89^**^	3266.7	3274.7	3294.4
HRS	.05^**^	.00	.43^**^	.03^**^	−.04^*^	.45^**^	22,280.4	22,308.4	22,407.7
Financial stressors									
LLLH	.03	.01	1.14^**^	.06	−.18	.90^**^	3234.2	3262.2	3331.3
HRS	.12^**^	−.00	.42^**^	.03^**^	−.04^*^	.44^**^	21,873.5	21,901.5	22,000.6
Spouse stressors									
LLLH	.03	.00	1.18^**^	.06	−.19	.85^**^	2366.6	2394.6	2459.3
HRS	.02^*^	.00	.33^**^	.02	−.00	.44^**^	14,539.6	14,567.6	14,661.0
Child stressors									
LLLH	.08^**^	−.03^*^	1.33^**^	.12^*^	−.28^**^	.82^**^	2950.5	2978.5	3046.3
HRS	.03^**^	−.00	.43^**^	.03^**^	−.03	.44^**^	20,478.8	20,506.8	20,604.9
Family stressors									
LLLH	.01	.00	1.33^**^	.11	−.27^**^	.83^**^	2984.6	3012.6	3080.5
HRS	.01	.00	.43^**^	.03^**^	−.03	.45^**^	20,682.3	20,710.3	20,808.5
Friend stressors									
LLLH	.11	−.02	1.21^**^	.08	−.22	.87^**^	3184.3	3214.3	3288.1
HRS	.01	.00	.46^**^	.04^**^	−.05^*^	.44^**^	20,640.4	20,688.4	20,766.5
PAIN INTERFERENCE									
Overall stressors									
LLLH	.02	.03	1.90^**^	.27^**^	−.59^**^	.32^**^	2717.1	2745.1	2814.1
HRS	.01^**^	.00	.05^**^	.01^**^	−.00	.09^**^	7474.2	7502.2	7601.4
Financial stressors									
LLLH	.02	.02	1.89^**^	.27^**^	−.59^**^	.31^**^	2705.2	2733.2	2802.3
HRS	.05^**^	−.00	.05^**^	.01^**^	−.00	.09^**^	7312.0	7340.0	7439.0
Spouse stressors									
LLLH	.00	.00	1.65^**^	.22^**^	−.47^**^	.29^**^	1978.2	2006.2	2070.9
HRS	.01^*^	.00	.04^**^	.00	−.00	.09^**^	4865.4	4893.4	4986.8
Child stressors									
LLLH	.03	.00	1.84^**^	.28^**^	−.58^**^	.30^**^	2492.6	2520.6	2588.4
HRS	.01^*^	.00	.06^**^	.01^**^	−.00	.09^**^	6923.7	6951.7	7049.8
Family stressors									
LLLH	−.02	.02	1.87^**^	.26^**^	−.57^**^	.31^**^	2505.7	2535.7	2608.3
HRS	.00	.01^**^	.06^**^	.01^**^	−.00	.09^**^	6984.6	7012.6	7110.7
Friend stressors									
LLLH	−.01	.02^*^	1.93^**^	.27^**^	−.59^**^	.31^**^	2843.2	2851.2	2870.9
HRS	.00	.00	.05^**^	.01^**^	−.00	.09^**^	6790.9	6820.9	6925.9

*Note.* LLLH = Longitudinal Late-Life Health sample; HRS = Health and Retirement Study sample; −2LL = −2 log likelihood; AIC = Akaike’s Information Criterion; BIC = Schwarz’s Bayesian Criterion.

All models adjusted for age, gender, and race.

* *p* < 0.05; ** *p* < 0.01.

With respect to effects of stressors on rates of change in number of painful conditions, in the HRS sample only, having higher overall stressor levels over an 8-year interval was associated with a small reduction in rate of increase (−.01, *p* < .05) in number of painful conditions over this interval. For these HRS participants, having more family stressors over the 8-year interval had a similar effect (−.01, *p* < .05) on rate of change in number of painful conditions.

Higher overall stressor levels, across 13-year (LLLH) and 8-year (HRS) intervals, were also associated with higher pain severity over these intervals (.11, *p* < .01 and .05, *p* < .01, respectively). With respect to individual stressor domains, elevations in child stressors were associated with more severe pain in both the LLLH (.08, *p* < .01) and HRS (.03, *p* < .01) samples. In the HRS sample only, heightened financial stressors and spouse stressors were also related to more severe pain (.12, *p* < .01 and .02, *p* < .05, respectively). Except in the domain of child stressors, which were associated with a reduced rate of increase in pain severity in the HRS sample (−.03, *p* < .01), stressor levels were unrelated to within-person rates of change in pain severity.

Stressors had some effects on pain interference but only in the HRS sample, where higher levels of overall, financial, spouse, and child stressors were associated with more pain interference (.01, *p* < .01; .05, *p* < .05; .01, *p* < .05.; and .01, *p* < .05, respectively). In the HRS sample only, heightened extended family stressors were associated with a slightly elevated within-person rate of increase in pain interference (.01, *p* < .01). In the LLLH sample only, elevated friend stressors were associated with a higher within-person rate of increase in pain interference (.02, *p* < .05).

### Gender and Race as Moderating Effects

Two statistically significant interactions between gender and stressors were found in the LLLH sample: For men more so than women, having more financial stressors was associated with having more painful conditions (−.12, *p* < .01). However, elevated financial stressors were associated with a slower rate of increase in number of painful conditions among men than women (.04, *p* < .05).

In the HRS sample, five statistically significant interactions showed that gender moderates the relationship between child stressors and levels and rates of change in pain. For women more so than men, higher levels of child stressors were associated with having more painful conditions, higher pain severity, and more pain interference (.03, *p* < 05; .03, *p* < .05; and .01, *p* < .05, respectively). However, elevated levels of child stressors were associated with a somewhat slower rate of increase in number of painful conditions and pain severity among women than among men (−.01, *p* < .05, for both relationships). Furthermore, for women more so than men, elevations in spouse stressors were associated with more pain interference (.02, *p* < .05).

There was only one statistically significant interaction between race and stressors on pain outcomes; it occurred in the HRS sample. For non-White more so than White participants, elevations in child stressors were linked to having more painful conditions (−.04, *p* < .05).

## Discussion

Although pain is often a salient feature of late-life, little is known about its longitudinal course, and its social environmental determinants, across the late-life span. Consistent with previous reports of cross-sectional age differences in pain (Abdulla et al., 2013; Fayaz et al., 2016), and the effects of age on repeated measures of pain (Fayaz et al., 2016), this study demonstrated that older adults experience significant within-person increases in pain across relatively long intervals during the late-life span.

This study also showed within-person declines in stressors over 13- and 8-year intervals, a finding consistent with earlier longitudinal research showing an association between older age and within-person declines in occurrence of stressful life events (Aldwin et al., 2011). In the HRS sample, these declines occurred in most of the individual stressor domains examined in this study, whereas in the LLLH sample they occurred only in the domains of children and extended family. Likely this difference occurred because the HRS sample was so much larger than the LLLH sample.

As predicted, in both the LLLH and HRS samples, older adults who reported higher overall stressor levels, across 13- and 8-year intervals, also experienced a larger number of painful conditions, and more severe pain, over these intervals. In the HRS sample, older adults with higher overall stressor levels also reported higher elevations in pain interference.

There were only a few, small associations between stressor levels and rates of change in pain in the LLLH and HRS samples. This may have occurred due to the relatively low average levels of pain, and minimal variance in its rate of change, during the 13- and 18-year time intervals covered in this investigation. Notably, among the associations found between stressors and rates of change in pain, most involved child- and extended family-related stressors, highlighting the salience of children and extended family in the dynamics of stressors and change in pain in later life.

Overall, the results of this study add to previous findings (Brennan, 2020) to show that stressors and pain are positively associated during the late-life span. However, due to the design of this study, which examined longitudinal stressor trajectories in relation to longitudinal pain trajectories, it is not possible to infer the direction of causality between stressors and pain in the LLLH and HRS samples. The positive associations between stressors and pain found here may reflect ongoing, reciprocal effects of stressors and pain that occur during later life, with elevated stressors eliciting and maintaining heightened pain, and pain adversely affecting older adults’ material and social contexts, reflected in heightened stressors in the domains of finances, spouse, children, extended family, and friends. This reciprocal relationship may be tightly bound temporally or even simultaneous: social and physical pain appears to share common neural substrates (Eisenberger, 2015; Tchalova & Eisenberger, 2015). This commonality may help explain why people with more exposure to early-life trauma report more physical pain in later life (Brown et al., 2005; Landa et al., 2012). The positive associations between stressors and pain shown here may also reflect shared activity in the neural substrate held in common by social and physical pain.

Notably, the positive associations between stressors and pain found here were often of relatively small magnitude, suggesting the need to search further for possible indirect physiological and psychological *mediating* pathways that might help explain the link between stressors and pain in later life. Physiological and psychological reactivity to stressors, including inflammatory, immunologic, emotional, and cognitive responses to stressors, may be key explanatory mechanisms that link late-life stressors and pain (Brennan, 2020).

Further inquiry should also be directed at the question of whether older adults’ personal and social resources are key *moderating* factors that affect the strength of the relationship between their stressors and pain. Previous research has shown that higher levels of socioeconomic advantage, sense of control, social support, resilience, and other personal and social resources can lessen the impacts of stressors on negative health outcomes, such as cardiovascular disease, diabetes, and depressive symptoms (e.g., Havnen et al., 2020; Holt-Lunstad, 2018; Infurna & Mayer, 2015; Lachman et al., 2011; Maisel & Karney, 2012; Thoits, 2010). These may also play critical roles in diminishing the strength of the relationship between late-life stressors and pain.

This study tested the *differential vulnerability hypothesis* (Aneshensel, 1992; Kessler, 1979) which, as applied to older adults’ stressors and pain, predicts that the strength of association between stressors and pain should be stronger among women than men and stronger among non-White than White persons. The few interactions that were statistically significant highlight their stressor domain specificity: Moderating effects of gender and race on the stressor-pain relationship occurred mainly where child stressors were elevated. This finding suggests that, compared to men and White persons, women and non-White persons may be more susceptible to experiencing elevations in pain in response to heightened child stressors. However, another interpretation is that this finding reflects gender- and race-based differences in intergenerational caregiving dynamics, such that women’s and non-White persons’ pain or pain-generating illness puts them into closer, more frequent, and potentially more stressor-ridden contact with their children than is the case for men’s and White persons’ pain or pain-generating illness.

In this investigation, harmonized data from two separate longitudinal studies of aging and health were analyzed to determine whether hypothesized relationships between stressors and pain found in one of the studies’ samples might be reproduced in the other. Broadly, regarding the presence, direction, and statistical significance of effects, findings predicted to occur in the LLLH sample were reproduced in the HRS sample. However, the HRS sample yielded a higher number of, and sometimes larger, effects than did the LLLH sample. Likely this was due to the larger HRS sample size. Thus, although the broad reproducibility demonstrated here enhances confidence in the existence of predicted relationships between late-life stressors and pain, it would be further bolstered by additional tests of reproducibility using, for example, harmonized longitudinal data from other large national studies closer in size to the HRS, such as the English Longitudinal Study of Ageing (ELSA; Steptoe et al., 2013).

This study had several limitations. As noted earlier, its design precludes inferences regarding direction of causality between stressors and pain across the late-life span. Moreover, its results generalize only to predominately White, “younger-old”, and community-residing participants who have provided three repeated measures of stressors, and of pain, over 13- and 8-year intervals. The measures of stressors and pain used in this study had drawbacks. For example, this study assessed participants’ stressors in only five life domains because these five stressor measures were the only ones available to harmonize across the two samples examined in this study. No doubt these five measures did not capture the full range of stressors experienced by participants. Similarly, limited measures of pain, and no measures of pain treatment, were used in this study. Both the LLLH and HRS parent investigations broadly assessed participants’ pain, but not its origins, body locations, or chronicity. It would be useful to know whether stressors impact these aspects of older adults’ pain experience and the receipt and effectiveness of their pain treatments.

This study also had several strengths, most notably use of two independent samples of older community residents, assessed with well-established, harmonized, repeated measures of stressors and pain, followed over time intervals of similar length. Its findings have potential clinical and public health implications. They suggest that older adults whose stressors are elevated and sustained during later life are at risk for corresponding elevations in number of painful conditions, pain severity, and more pain interference. Screening procedures might be employed to identify older adults with these sustained stressor elevations, and to target them for interventions to diminish associations between their stressful circumstances and pain experiences.

In conclusion, this investigation adopted a longitudinal, life course perspective on stressors and pain to demonstrate that, across the late-life span, older adults’ stressors and pain are linked. The reasons why, and the direction of causality in this association, remain to be determined. Future research should focus on the mediating mechanisms that account for the association between late-life stressors and pain, and the moderating factors that affect its strength. Such work will contribute to further elaboration and expansion of the biopsychosocial model of pain, which posits that pain is a product of complex interactions among biological, psychological, and social factors. Further, it may help guide development of effective clinical and public health approaches aimed at preventing and weakening links between late-life stressors and pain.
